# Supplementary material for: Effect of Ciprofloxacin on the Composition of Intestinal Microbiota in Sarcophaga peregrina (Diptera: Sarcophagidae)
Source: Microorganisms. 2023 Nov 27;11(12):2867. doi: 10.3390/microorganisms11122867 (PMC10745613; doi:10.3390/microorganisms11122867)

Figure S1 Boxplot analysis of the relative abundance of the top 10 at different levels.

(a) phylum **(b)** genus.

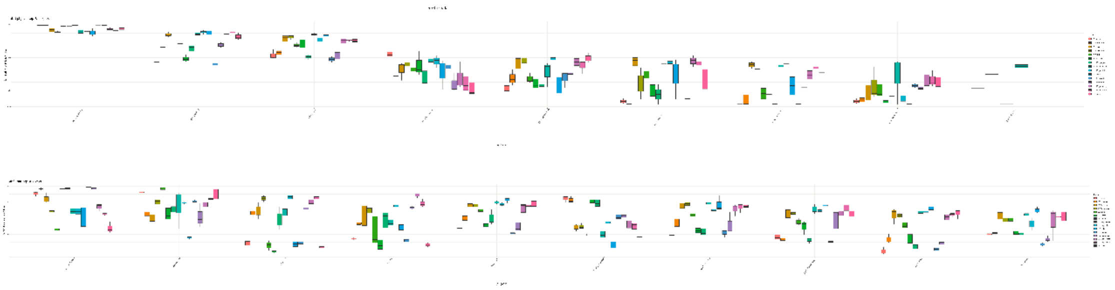

Figure S2 Line charts of the relative abundance of the Top 10 at the genus level. **(a)***Halomonas*, **(b)***Escherichia-Shigella*, **(c)***Muribaculaceae*, **(d)** *Prevotella*.

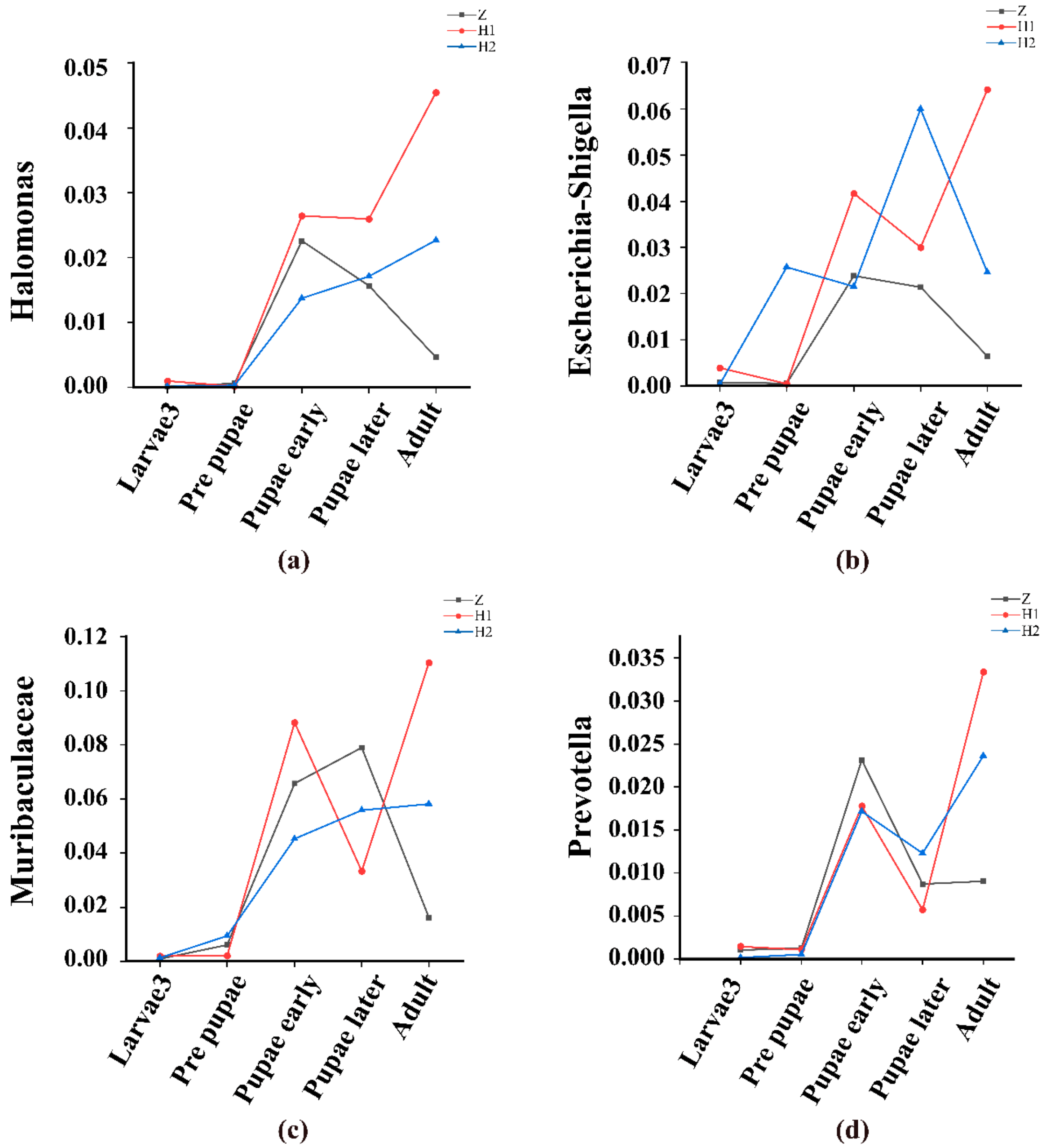

Supplement: Supplementary file 1 [file microorganisms-11-02867-s001.zip › Supplementary Data File S1.pdf]
